# Variability of risk factors and diabetes complications

**DOI:** 10.1186/s12933-021-01289-4

**Published:** 2021-05-07

**Authors:** Antonio Ceriello, Francesco Prattichizzo

**Affiliations:** IRCCS MultiMedica, Via Gaudenzio Fantoli, 16/15, 20138 Milan, Italy

**Keywords:** Diabetes mellitus, Glucose variability, Blood pressure variability, Lipids variability, Body weight variability, Uric acid variability, Heart rate variability, Oxidative stress, Cardiovascular complications, Microvascular complications

## Abstract

Several studies suggest that, together with glucose variability, the variability of other risk factors, as blood pressure, plasma lipids, heart rate, body weight, and serum uric acid, might play a role in the development of diabetes complications. Moreover, the variability of each risk factor, when contemporarily present, may have additive effects. However, the question is whether variability is causal or a marker. Evidence shows that the quality of care and the attainment of the target impact on the variability of all risk factors. On the other hand, for some of them causality may be considered. Although specific studies are still lacking, it should be useful checking the variability of a risk factor, together with its magnitude out of the normal range, in clinical practice. This can lead to an improvement of the quality of care, which, in turn, could further hesitate in an improvement of risk factors variability.

## Background

Growing attention has been recently paid to the possible the role of glucose variability (GV) in the development of diabetic complications, particularly cardiovascular (CV) ones [1].

Many observational [1] and some interventional studies [2], as well as post-hoc analyses of trials such as the “Action in Diabetes and Vascular Disease: Preterax and Diamicron MR Controlled Evaluation” (ADVANCE), [3] the “Trial Comparing Cardiovascular Safety of Insulin Degludec vs. Insulin Glargine in Patients With Type 2 Diabetes at High Risk of Cardiovascular Events” (DEVOTE), [4] the “Veterans Affairs Diabetes Trial” (VADT), [5] the “Antihypertensive and Lipid-Lowering Treatment to Prevent Heart Attack Trial” (HALLHAT), [6] the “Action to Control Cardiovascular Risk in Diabetes” (ACCORD) trial, [7] and the “Empagliflozin Cardiovascular Outcome Event (EMPA-REG OUTCOME) trial [8] confirm that in type 2 diabetes (T2D) long-term GV is correlated with an increased risk of both CV and microvascular complications. However, we need to underline that while we pay much attention to GV, [1] emerging evidence suggests that also the long-term variability of other risk factors may be involved in the development of diabetes complications.

Long-term variability is defined as the fluctuations of a certain risk factor outside the recommended range in successive measures [1]. While a number of metrics has been proposed to assess long-term variability, the most commonly used is the standard deviation (SD) of the collected values [1].

Common risk factors for the development of diabetes complications include blood pressure, lipid parameters (i.e. total, HDL-, and LDL-cholesterol and triglycerides), heart rate, body weight, and uric acid. Here we summarize the key available literature describing the variability of such risk factors in relation to the development of complications in patients with diabetes. Then, we synthesize the therapeutic options that seem available at the moment to face this new emerging challenge of diabetes management.

## Variability of risk factors and diabetic complications

### Blood pressure

Grove et al. [9] originally observed variability in visit-to-visit systolic blood pressure (SBP) was related to incidence of coronary heart disease in a population with or without hypertension.

Several studies, after this finding, have reported that a blood pressure (both systolic and diastolic) variability in people with diabetes is an independent predictor of both macro- and microvascular complications, particularly nephropathy.

The ADVANCE was a factorial randomized controlled trial to test the effect of a tight control of both glucose and blood pressure in patients with T2D on major adverse cardiovascular events (MACE), i.e. myocardial infarction, stroke, or CV death and microvascular endpoints, i.e. new or worsening nephropathy or retinopathy [10]. SBP was measured at six successive visits for 24 months after randomization and was used to estimate its variability, defined as SD. During a median 2.4 years of follow-up, the SD of SBP variability was associated with the incidence of both MACE and microvascular events despite multiple adjustments for a plethora of variables including mean SBP [10].

The ADVANCE-ON (ADVANCE-Observational) followed-up 9114 patients not experiencing MACE, renal events, or death during the active phase of the trial for an additional observational follow-up of 7.6 years after termination of the treatments. The SD of SBP, measured during the 24 months of active phase of the trial, was log-linearly and independently associated with an increased risk of the primary outcome, i.e. MACE, renal events, or death, during the protracted follow-up, extending the previous findings to a longer-term range [11].

A retrospective cohort study was conducted in primary care and analyzed 124,105 Chinese adults with T2D and without prior diagnosed CV disease (CVD) [12]. During a median follow-up of 39.5 months, a positive linear relationship was observed between the SBP variability and the incidence of both newly developed CVD and all-cause mortality, irrespectively of the mean SBP [12]. In particular, patients with an SD of SBP of < 5 mmHg had the lowest risks while patients with an SD of ≥ 10 mmHg had the highest risk [12]. Another, retrospective cohort study enrolling 10,163 patients with T2D and free of CVD at baseline showed that the variability of SBP was associated with an increased risk of CVD, independently of the mean SBP level [13]. Of note, five different metrics, i.e. SD, coefficient of variation, variation independent of mean, average real variability, and successive variability of measurements, measured during 24-months of observation, were used to perform the analysis [13]. In the “The Rio de Janeiro Type 2 Diabetes Cohort Study”, SBP-visit-to-visit variability emerged as an independent predictor of MACE (hazard ratio: 1.25, 95% CI 1.03–1.51 for a 1-SD increase in 24-month SD), but not of total CV events, CV and all-cause mortality, and of any microvascular outcome [14].

Very recently, pooled data from the ACCORD and VADT Trials have shown that both systolic and diastolic blood pressure variability can favor the development of heart failure in T2D, an effect likely mediated by dips, not elevations, of blood pressure [15].

A post-hoc analysis pooling data from the Irbesartan Diabetic Nephropathy Trial (IDNT) and the “Reduction of End Points in Non-Insulin-Dependent Diabetes With the Angiotensin II Antagonist Losartan” (RENAAL) Study explored the effect of long-term blood pressure variability on CV and renal endpoints in 2739 participants with T2D and nephropathy [16]. The renal endpoint was a composite of time to confirmed doubling of serum creatinine level, development of end-stage renal disease, or death, while the CV outcome was defined as CV death, myocardial infarction, stroke, hospitalization for heart failure, or revascularization [16]. Larger SBP visit-to-visit variability was independently associated with an increased risk for the composite renal endpoint, but not with the CV outcome [16]. Similarly, another study taking advantage of clinical records from 30,851 T2D patients with hypertension and normal estimated glomerular filtration rate (eGFR) at baseline found that an increased long-term blood pressure variability predicted kidney disease, defined as a composite of eGFR less than 60 and/or a decrease in eGFR at least 30% from baseline levels during a 4-year follow-up [17]. Of note, SBP variability was measured by three metrics: coefficient of variation, SD of the mean SBP and average absolute difference of successive values in each individual, all providing similar results [17]. Several clinical characteristics (older age, male sex, SBP, diastolic blood pressure, albuminuria, glycated hemoglobin, insulin treatment) were related to intra-individual SBP variability [17]. More recently, the role of visit-to-visit variability of blood pressure on the development of hypertension and changes in renal function in patients with T2D diabetes and normal blood pressure has been evaluated in a real-life clinical setting [18]. After a mean follow-up time of 3.5 ± 2.8 years, an increase of 5 mmHg of visit-to-visit variability of blood pressure was associated with a 19% (P < 0.0001) and a 5% (P = 0.008) independent increased risk of developing hypertension and worsening of albuminuria, respectively [18].

Overall, a number of studies suggests that variability of SBP is independently associated with the development of a range of diabetes complications, while less data are available for diastolic blood pressure variability. The studies showing an association of blood pressure variability with diabetes complications are summarized in Table 1.

**Table 1:** Summary of the studies showing an effect of blood pressure variability on the development of complications in patients with diabetes

Risk factor	Type of variability assessed	Short or long term variability	Metrics used	Type of study	Sample size	Significantly associated outcomes	Follow-up length	References
Systolic blood pressure	Visit-to-visit	Long-term	Standard deviation	Post-hoc analysis of trial	9114	MACE; microvascular outomes	2.4 years	[10]
				Post-hoc analysis of trial	9114	MACE, renal events, or death;	7.6 years	[11]
				Retrospective cohort study	124105	Newly developed CVD; all-cause mortality	39.5 months	[12]
				Retrospective cohort study	10163	Risk of CVD	24 months	[13]
				Prospective cohort study	632	MACE	11.3 years	[14]
				Post-hoc analysis of 2 trials	2739	Composite renal outcome	2.6/3.4 years	[16]
				Retrospective cohort study	30851	Composite renal outcome	4 years	[17]
			Coefficient of variation	Post-hoc analysis of 2 trials	9383 plus 1550	Heart failure development	56.6 months/59.5 months	[15]
				Retrospective cohort study	10163	Risk of CVD	24 months	[13]
				Retrospective cohort study	30851	Composite renal outcome	4 years	[17]
			Variation independent of mean	Retrospective cohort study	10163	Risk of CVD	24 months	[13]
			Average real variability	Retrospective cohort study	10163	risk of CVD	24 months	[13]
				Post-hoc analysis of 2 trials	9383 plus 1550	Heart failure related event	56.6 months/59.5 months	[15]
				Retrospective cohort study	30851	Composite renal outcome	4 years	[17]
			Successive variability of measurements	Retrospective cohort study	10163	Risk of CVD	24 months	[13]
Diastolic blood pressure	Visit-to-visit	Long-term	Coefficient of variation AND Average real variability	Post-hoc analysis of 2 trials	9383 plus 1550	Heart failure development	56.6 months/59.5 months	[15]

### Lipids

High-density-lipoprotein-cholesterol (HDL), triglycerides, and low-density-lipoprotein-cholesterol (LDL) visit-to-visit variability has been associated with CVD in non-diabetic subjects [19].

Patients with diabetes are known to suffer from a marked variability of the lipids plasma levels [20]. More recently, in 162 T2D patients followed for 1 year, it has been shown that the LDL variability was as independent determinant of carotid maximum IMT, independently of a number of variables including mean LDL levels, body mass index (BMI), waist circumference, diabetes therapy and duration, means and SD of glycaemic and other lipid variables, as well as the use of hypolipidemic and anti-hypertensive drugs [21]. A more recent study correlates high LDL variability with an increased risk of CVD in T2D [22].

In another study, data from 1792 subjects who underwent percutaneous coronary intervention were analyzed [23]. During a median follow-up of 65 months, 114 patients (6.4%) had a MACE [23]. Visit-to-visit variability in LDL-, HDL-, and non-HDL-cholesterol was significantly higher in the group suffering a CV event compared to those not experiencing the endpoint [23]. In the multiple regression analysis, LDL-, HDL-, and non-HDL-cholesterol variability parameters were all independent predictors of CV events after adjusting for a number of confounding variables. These relationships were also observed in the subgroup with diabetes [23].

Of note, HDL variability has been reported as risk factor for the appearance also of albuminuria in T2D [24]. Finally, while there are no data linking visit-to-visit variability of triglycerides to macrovascular complications of diabetes, two studies show that both high levels of postprandial (i.e. short-term variability) and visit-to-visit variability of triglycerides predict the development or worsening of nephropathy in diabetes [25–27]. The main characteristics of the studies showing an association of lipids variability with diabetes complications are summarized in Table 2.

**Table 2:** Summary of the studies showing an effect of lipids variability on the development of complications in patients with diabetes

Risk factor	Type of variability assessed	Short or long term variability	Metrics used	Type of study	Sample size	Significantly associated outcomes	Follow-up length	References
LDL	Visit-to-visit	Long-term	Standard deviation	Observational cohort	162	Carotid intima-media thickness	1 year	[21]
				Observational cohort	5354	Cardiovascular events	3.2 years	[22]
				Observational cohort	1792	Cardiovascular events	65 months	[23]
HDL	Visit-to-visit	Long-term	Standard deviation	Observational cohort	1792	Cardiovascular events	65 months	[23]
				Observational cohort	864	Appearance of albuminuria	3 years	[24]
Non-HDL cholesterol	Visit-to-visit	Long-term	Standard deviation	Observational cohort	1792	Cardiovascular events	65 months	[23]
Triglycerides	Visit-to-visit	Long-term	Standard deviation	Observational cohort	457	Incident microalbuminuria	6.8-years	[27]
	Post-prandial	Short-term	Coefficient of variation	Observational cohort	168	eGFR decline	6.0 years	[26]

### Body weight

Several years ago, the evidence that variability in body weight (BW) could be related to high risk of CVD emerged from the Framingham Study [28].

Data from three clinical trials were pooled and used to evaluate the impact of BW variability in 6408 patients with T2D on the development of macrovascular endpoints, using a composite of coronary heart disease death, myocardial infarction, resuscitated cardiac arrest, coronary revascularization, and unstable or new-onset angina as the primary endpoint [29]. When used as a time-dependent covariate, BW variability, measured as average successive variability, was linearly and independently associated with an increased risk of any coronary event, major coronary event, any CV event, and death. In particular, when comparing the highest with the lowest quintile of BW variability, the increased risk for any component of the composite outcome was substantially higher [29]. These results suggest that among subjects with T2D, fluctuation in BW is associated with higher mortality and a higher rate of CV events, independent of traditional CV risk factors [29].

The ACCORD trial participants’ weight was documented annually during the trial [30].

Out of the 10,251 ACCORD participants, 911 (8.9%) has normal weight, 2985 (29.1%) were overweight, and 6355 (62%) were obese. During a mean of 3.5 years of follow-up, BW variability was associated with the primary outcome MACE, but also with heart failure, death, and microvascular events, an observation independent of CV risk factors and BMI [30].

The “Verona Diabetes Study” explored the impact of variability of fasting glycaemia, BMI, and pulse pressure, measured as coefficient of variation, on all-cause mortality in 1319 subjects with T2D followed for 10 years [31]. When analyzing data according to age subgroups, the variability of glycaemia, BMI and pulse pressure independently predicted all-cause mortality in patients > 65 years, but not in younger subjects, suggesting a possible role for the variability of these risk factors in determining mortality in older patients with T2D [31].

### Uric acid

High level of uric acid has been reported to be a risk factor for both CVD and nephropathy in diabetes [32, 33].

There are not specific studies relative to uric acid variability in relation to CVD development in patients with diabetes. However, in 8822 non-diabetic men, aged 40–65, followed for 5 years, coronary heart disease and all-cause mortality yielded a significant association with the variability of uric acid [34]. More recently, high uric acid variability has been found associated with a higher risk of developing future CV events in patient with coronary artery disease [35].

A recent study showed that the variability of uric acid was predictive of kidney alteration, particularly of eGRF decline, in T2D [36]. Of note, serum uric acid levels are related to insulin resistance and BMI, rather than insulin levels, suggesting that obesity-driven metabolic syndrome as a major determinant of its levels [37].

### Heart rate

Differently from the other discussed risk factors, heart rate (HR) variability is often studied by a resting ECG or with a 24-h Holter, thus it refers to short-term variability and not to visit-to-visit variability. In particular, low-frequency (0.04–0.15 Hz) oscillations are a surrogate measure of HR variability ascribable to both sympathetic and parasympathetic innervation [38–41]. Cardiac autonomic neuropathy affects the parasympathetic nervous system, leading to reduced HR variability [38]. In people without diabetes, decreased HR variability is associated with an increased incidence of cardiac events [39].

Low HR variability has been reported in both type [1] (T1D) and T2D and, intriguingly, in pre-diabetes [40].

A preliminary study explored the impact of autonomic neuropathy on HR variability and on the progression of atherosclerosis in 61 T2D patients followed for 8 years [41]. Results suggested that patients with autonomic neuropathy had decreased low frequency HR variability, which was correlated with both a reduced carotid artery diameter and an increased atherosclerotic intima-media thickness (IMT). HR variability further decreased during follow-up, while patients with lower frequency of HR at baseline had a more relevant enlargement in the thickness of the carotid bulb intima-media at follow-up, possibly suggesting that a low frequency HR variability might foresee the degree of atherosclerosis progression in patients with T2D [41].

Subjects with T1D or those without diabetes from the “Coronary Artery Calcification in Type 1 Diabetes” study underwent supine deep breathing 12-lead electrocardiograms [42]. The SD of consecutive RR intervals was used as a measure of HR variability. Coronary artery calcium was measured at two visits 6 years apart. Reduced HR variability was associated in both T1D and healthy controls with older age, higher HbA1c, elevated albuminuria, coronary artery calcium volume, as well as higher fibrinogen levels at baseline. Higher HR variability at baseline was independently associated with a decreased probability of coronary artery calcium progression, even after adjusting for a range of CV risk factors including inflammatory parameters [42].

Finally, two studies, both with a follow-up of about 5 years, were able to show that low HR variability is related to a high risk of cardiac and total mortality in T2D [43, 44]. The main characteristics of the studies showing an association between body weight, uric acid, and heart variability and diabetes complications are summarized in Table 3.

**Table 3:** Summary of the studies showing an effect of body weight, uric acid, and heart rate variability on the development of complications in patients with diabetes

Risk factor	Type of variability assessed	Short or long term variability	Metrics used	Type of study	Sample size	Significantly associated outcomes	Follow-up length	References
Body weight	Visit-to-visit	Long-term	Average successive variability	Post-hoc analysis of 3 trials	6408	Composite of cardiovascular events	3.9/4/4.9 years	[29]
				Post-hoc analysis of trial	10251	MACE, heart failure, death, and microvascular events	3.5 years	[30]
			Coefficient of variation	Observational cohort	1319	All-cause mortality in patients > 65 years	10 years	[31]
Uric acid	Visit-to-visit	Long-term	Standard deviation	Retrospective cohort study	10163	eGRF decline	24 months	[36]
Heart rate	24-h ECG Holter, expiration/inspiration (E/I) ratio during deep breathing, acceleration index (AI) of R-R interval in response to head-up tilt	Short-term	Low frequency	Observational cohort	61	Intima-media thickness	8 years	[41]
	Supine deep breathing 12-lead electrocardiograms	Short-term	Standard deviation of consecutive RR intervals	Observational cohort	1416	Coronary artery calcium	6.0 ± 0.5 years	[42]
			Coefficient of variance for 100 R–R intervals	Observational cohort	8917 (3089 with diabetes)	Sudden cardiac death	5.2 years	[43]
	24-h ECG Holter	Short-term	Standard deviation of NN intervals	Observational cohort	240	All-cause mortality	15.5 years	[44]

## Interaction between the variability of the risk factors

It is well known that there is a cumulative effect of the various risk factors in producing serious CV complications. This evidence originally came out from the historical Framingham Heart Study [45]. In the same study it was also clear that the presence of diabetes contributes to amplify the additive effect of the risk factors in the development of the CV complications [45].

As above reported, the variability of a risk factor contributes an additive risk, independently from the magnitude and the duration of the abnormal level of the risk factor. The research in this field is relatively new, however, some evidence already exists. Studies have explored the effect of the simultaneous variability of several risk factors on a target complication. In non-diabetic subjects the combined variability of several risk factors contributes to the risk for both CVD and end stage renal disease [46, 47]. An additive effect of HbA1c and blood pressure variability on the risk of mortality has been reported in T1D patients followed-up for 1430 days [48]. The hazard ratio for high HbA1c variability was 1.78 ± 0.36. The hazard ratio for high SBP variability was 1.69 ± 0.33. The hazard ratio for high HbA1c and high SBP variability together was 2.37 ± 0.32 [48]. In older T2D patients the variability of fasting glycaemia, BW and blood pressure were independently associated with an increased risk of all-cause mortality during the 10 years of follow-up [31]. In people with T2D an inverse association has been reported between glucose fluctuations and heart rate variability [49]. Another study reported that glucose and blood pressure variability were associated with endothelial and CV damage in diabetic patients with optimal metabolic control [50]. However, in a population of patients with T2D and no history of CVD, in which other CV risk factors were within or near to the recommended targets, only 2-h post breakfast blood glucose level, but not the variability of blood pressure, lipids and creatinine, was associated with incident CVD, as observed during the follow-up period of 5–8 years [51].

In T2D the additive effect of HbA1c, fasting glucose, systolic and diastolic blood pressure, total-cholesterol, HDL, LDL, triglycerides, and uric acid variability on the appearance of kidney disease has been reported [36].

**Fig. 1:**
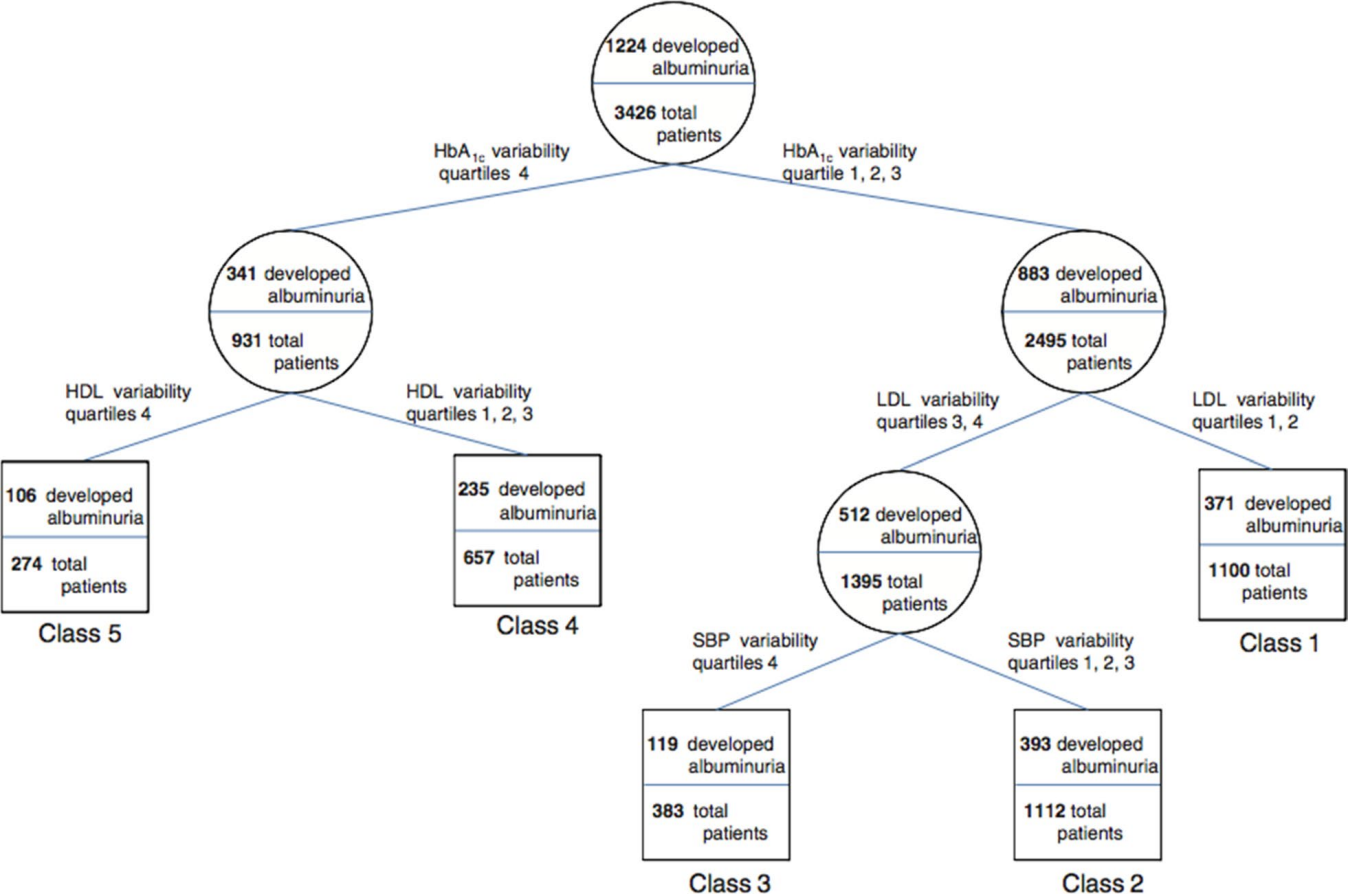
Recursive partitioning techniques (RECPAM) analysis of developing albuminuria in a cohort of 4231 patients with T2D followed up for a median of 3.4 years and with 5 subsequent measurements of risk factors [36]. The tree-growing algorithm resumes the hazard of developing albuminuria according to a multivariable Cox regression analysis. At each step, the method proceeds forward using the covariate with the highest difference in risk. The algorithm proceeds until user-defined conditions are met. Variables used to build the model were quartiles of variability in HbA1c, systolic blood pressure (SBP) and diastolic blood pressure (DBP), serum uric acid (UA), total, high-density lipoprotein (HDL), low-density lipoprotein (LDL) cholesterol and triglycerides., while additional baseline parameters were considered in the model as global variables, i.e. age, gender, duration of diabetes, smoking, hypertension, baseline HbA1c, blood pressure, UA, lipid parameters and estimated glomerular filtration rate (eGFR) values. The variable determining patient’s assignment to the subsequent group is evidenced on the branch proceeding to the following subgroup, while rectangles represent the REPCAM class. The numbers in the circles and rectangles represent the patients who develop albuminuria compared with the total number of patients in the subgroup, respectively (Reproduced with permission from Ref. [36])

**Fig. 2:**
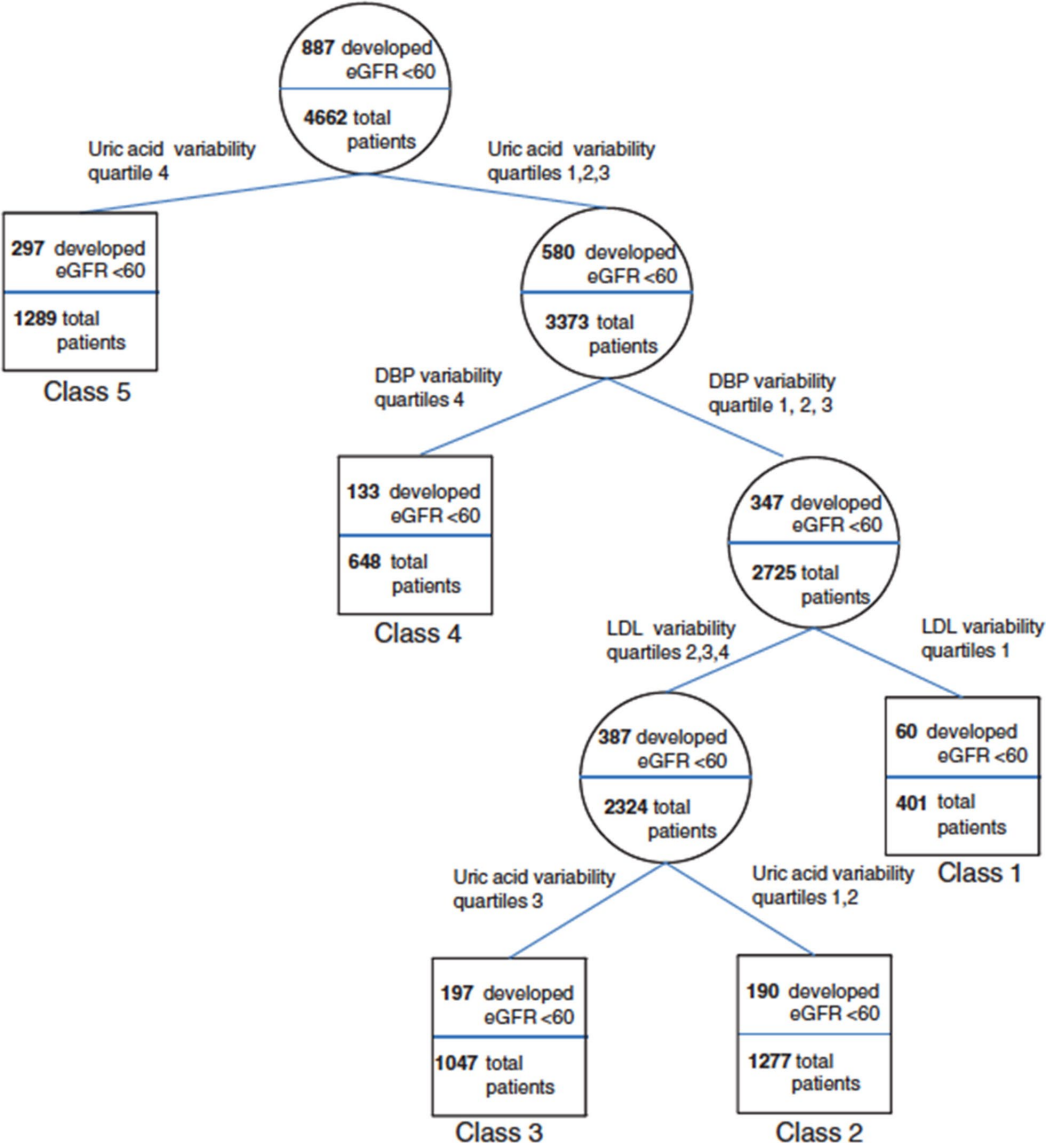
RECPAM analysis of developing a decrease in glomerular filtration rate (GFR). The RECPAM tree-growing algorithm models the hazard of developing GFR < 60 mL/min/1.73 m^2^ using the same approach and the same variables described for Fig. 1 in the same population (Reproduced with permission from Ref. [36])

A recent study using data from a clinical database reporting at least 5 measures of multiple risk factors from 4231 patients with T2D followed up for a median of 3.4 years showed that a significantly higher risk of developing albuminuria was associated with variability in HbA1c, while the variability in systolic and diastolic blood pressure, HDL, LDL and uric acid predicted the decline in eGFR, with the association with uric acid variability being particularly strong [36]. In addition, the concomitance of high variability in HbA1c and HDL conferred the highest risk of developing albuminuria (Fig. 1), while a high variability in uric acid or diastolic blood pressure conferred the highest risk of decline in eGFR (Fig. 2). This novel evidence suggests that the contribution of the variability of each single factor might have higher or lesser impact according to the specific complication studied [36]. This new field of research needs more studies.

## Is it time to treat?

The major issue is whether risk factors variability is causal or a marker, and, while with glucose the case for causality can be made [1], it would be more difficult with uric acid, for example.

The evidence in favour to be just a marker is that the overall quality of care seems conditioning the variability of all these risk factors [52]. The quality-of-care summary score (Q-score) is a surrogate, validated measure of quality of care as a whole. The frequency of risk factors measurement, their values, and the relative pharmacological therapy contribute to produce the Q-score, the values of which can range between zero and forty with higher scores being descriptive of better quality of care [52]. In a study including 273,888 people with diabetes the variability of HbA1c, systolic and diastolic blood pressure, total cholesterol, LDL, HDL, and uric acid was inversely correlated with the Q-score value [52]. In a multivariate linear regression analysis a Q-score > 25 was associated with a significantly minor variation in HbA1c, systolic and diastolic blood pressure, uric acid, total cholesterol, HDL, and LDL cholesterol, when compared to a score < 15 [52]. The analysis of standardized β coefficients evidenced that the Q-score had a higher impact on the variability of HbA_1c_, SBP, total cholesterol, and LDL cholesterol [52]. This finding suggests that when a quality-of-care improvement is pursued to increase the achievement of the recommended targets, this action might be accompanied by a reduction of risk factors variability. This seems to be the case. In a recent study, the attainment of HbA1c, blood pressure, and LDL-cholesterol goals was associated with a significant improvement of their variability [53].

However, it also true that, according to Hill definition of causality, the variability of several risk factors does fit with this concept [54]. Indeed, the findings relative to the association with the quality of care intuitively suggest that risk factors variability might result from clinical inertia and drug non-adherence, two pervasive phenomena in real-world settings of diabetes treatment [55]. Therefore, more studies are needed to explore both the relevance of these two phenomena in determining variability and to assess if variability of risk factors per se represents an additive driver of diabetes complications.

The current strategy for the management of a risk factor is to try to maintain its levels within the normal range as long as possible. The evidence that the variability in time of a risk factor has a potential harmful effect on the development of diabetic complications opens the discussion of whether a new therapeutic strategy is needed: the normalization of the level of a risk factor might be not enough without reducing its variability. Whether it seems more than acceptable to improve the quality of care and to reach the optimal target, hoping that these actions may also improve the variability of the risk factors [52, 53], the key issue is whether it is also the case to plan more specific interventions to improve the variability of a risk factor. This seems, for example, the case for glucose variability [56].

The question is certainly not only a scientific curiosity. There is also the possibility of intervening on the variability of a specific risk factor.

Diverse anti-hypertensive drugs have different effects on reducing blood pressure variability. Indeed, a meta-analysis suggested that variability of SBP was decreased by calcium-channel blockers and thiazides, while it was increased by angiotensin-converting enzyme inhibitors, angiotensin-receptor blockers, and beta-blockers [57]. Similarly, the use of a SGLT-2 inhibitor may also be helpful in reducing not only blood pressure but also its variability [58].

Although statin therapy is widely known to substantially decrease LDL, variability in LDL levels during the treatment has been observed. Although visit-to-visit variability in LDL is largely attributable to statin non-adherence, it is worthy to remember that Bangalore et al. showed that LDL variability was consistently less pronounced with the atorvastatin dose of 80 mg/day compared to the 10 mg/day dose [59, 60].

Moderate weight loss has been preliminary reported to improve HR variability in T2D [61]. Finally, weight loss strategies that minimize weight cycling probably should be given preference over those that are prone to cause a “yoyo effect” [62].

## Conclusions

In most of the intervention studies in settings of T2D, a reduction in the magnitude of diverse CV risk factors have translated into tangible benefits on hard endpoints. On the other side, no trial has clearly proven that a reduction in variability per se, independently of the risk factor magnitude, is beneficial. However, evidence is growing suggesting that the variability of a risk factor might be not less dangerous than its magnitude and/or the time spent having it in an abnormal level. Moreover, it is also enough clear that the variability of each factor is additive in leading to the final picture of a diabetic complication. Therefore, from a therapeutic viewpoint, is it time to try to reduce both magnitude and variability of CV risk factors? Because an improvement of the quality of care [52] and the attainment of their target [53] seem to be the best strategy to reach this goal, we believe that the answer is not complicated.

## Data Availability

Not applicable.
